# Globally Optimal Multisensor Distributed Random Parameter Matrices Kalman Filtering Fusion with Applications

**DOI:** 10.3390/s8128086

**Published:** 2008-12-08

**Authors:** Yingting Luo, Yunmin Zhu, Dandan Luo, Jie Zhou, Enbin Song, Donghua Wang

**Affiliations:** Department of Mathematics, Sichuan University, Chengdu, Sichuan, 610064, P. R. China; E-Mail: lyt83@163.com

**Keywords:** Random parameters matrices, Kalman filtering, Centralized fusion, Distributed fusion

## Abstract

This paper proposes a new distributed Kalman filtering fusion with random state transition and measurement matrices, i.e., random parameter matrices Kalman filtering. It is proved that under a mild condition the fused state estimate is equivalent to the centralized Kalman filtering using all sensor measurements; therefore, it achieves the best performance. More importantly, this result can be applied to Kalman filtering with uncertain observations including the measurement with a false alarm probability as a special case, as well as, randomly variant dynamic systems with multiple models. Numerical examples are given which support our analysis and show significant performance loss of ignoring the randomness of the parameter matrices.

## 1. Introduction

Linear discrete time system with random state transition and observation matrices arise in many areas such as radar control, missile track estimation, satellite navigation, digital control of chemical processes, economic systems. Koning [1] gave the Linear Minimum Variance recursive estimation formulae for the linear discrete time dynamic system with random state transit and measurement matrices without detailed rigorous derivation. Such system can be converted to a linear dynamic system with deterministic parameter matrices and state-dependent process and measurement noises. Therefore, the conditions of standard Kalman Filtering are violated and the recursive formulae in [1] can not be derived directly from the Kalman Filtering Theory. In this paper, a rigorous analysis (mainly in the appendix) shows that under mild conditions, the converted system still satisfies the conditions of standard Kalman Filtering; therefore, the recursive state estimation of this system is still of the form of a modified Kalman filtering. Reference [5] shows that this result can be applied to Kalman filtering with uncertain observations, as well as randomly variant dynamic systems with multiple models.

Many advanced systems now make use of large number of sensors in practical applications ranging from aerospace and defense, robotics and automation systems, to the monitoring and control of a process generation plants. An important practical problem in the above systems is to find an optimal state estimator given the observations.

When the processing center can receive all measurements from the local sensors in time, centralized Kalman filtering can be carried out, and the resulting state estimates are optimal in the Mean Square Error (MSE) sense. Unfortunately, due to limited communication bandwidth, or to increase survivability of the system in a poor environment, such as a war situation, every local sensor has to carry on Kalman filtering upon its own observations first for local requirement, and then transmits the processed data-local state estimate to a fusion center. Therefore, the fusion center now needs to fuse all received local estimates to yield a globally optimal state estimate.

Under some regularity conditions, in particular, the assumption of independent sensor noises, an optimal Kalman filtering fusion was proposed in [11-12], which was proved to be equivalent to the centralized Kalman filtering using all sensor measurements; therefore, such fusion is globally optimal. Then, Song [7] proved that under a mild condition the fused state estimate is equivalent to the centralized Kalman filtering using all sensor measurements.

In the multisensor random parameter matrices case, sometimes, even if the original sensor noises are mutually independent, the sensor noises of the converted system are still cross-correlated. Hence, such multisensor system seems not satisfying the conditions for the distributed Kalman filtering fusion given in [11-12]. In this paper, it was proved that when the sensor noises or the random measurement matrices of the original system are correlated across sensors, the sensor noises of the converted system are cross-correlated. Even if so, similarly with [7], centralized random parameter matrices Kalman filtering, where the fusion center can receive all sensor measurements, can still be expressed by a linear combination of the local estimates. Therefore, the performance of the distributed filtering fusion is the same as that of the centralized fusion under the assumption that the expectations of all sensor measurement matrices are of full row rank. Numerical examples are given which support our analysis and show significant performance loss of ignoring the randomness of the parameter matrices.

The remainder of this paper is organized as follows. In Section 2, we present the concept of random parameter matrices Kalman filtering. In Section 3, we present an optimal Kalman filtering fusion with random parameter matrices and show that under a mild condition the fused state estimate is equivalent to the centralized Kalman filtering with all sensor measurements. In Section 4, we show that the result can be applied to Kalman filtering with uncertain observations as well as randomly variant dynamic systems with multiple models. More importantly, we will see that the Kalman filtering with false alarm probability is a special case of Kalman with random parameter matrices. A simulation example is given in Section 5. And finally, in Section 6, we present our conclusions.

## 2. Random Parameter Matrices Kalman Filtering

Consider a discrete time dynamic system:

(1) $$x_{k+1}=F_{k}x_{k}+v_{k}$$

(2) $$y_{k}=H_{k}x_{k}+\omega_{k},\;k=0,1,2,\ldots$$

where *x_k_* ∈ *R^r^* is the system state, *y_k_* ∈ *R^N^* is the measurement matrix, *v_k_* ∈ *R^r^* is the process noise, and *ω_k_* ∈ *R^N^* is the measurement noise. The subscript *k* is the time index. *F_k_* ∈ *R^r^*^×^*^r^* and *H_k_* ∈ *R^N^*^×^*^r^* are random matrices.

We assume the system has the following statistical properties: {*F_k_*, *H_k_*, *v_k_*, *ω_k_*, *k* = 0,1,2,…} are all sequences of independent random variables temporally and across sequences as well as independent of *x*_0_. Moreover, we assume *x_k_* and {*F_k_*, *H_k_*, *k* =0,1,2,…} are mutually independent. The initial state*x*_0_, the noises *v_k_*, *ω_k_*and the parameter matrices *F_k_*, *H_k_* have the following means and covariance.

(3) $$E(x_{0})=\mu_{0},E(x_{0}-\mu_{0}){\left(x_{0}-\mu_{0}\right)}^{T}=P_{0},$$

(4) $$E(v_{k})=0,E(v_{k}v_{k}^{T})=R_{v_{k}},E(\omega_{k})=0,E(\omega_{k}\omega_{k}^{T})=R_{\omega_{k}}$$

(5) $$E(F_{k})={\bar{F}}_{k},\text{Cov}(f_{ij}^{k},f_{mn}^{k})=C_{f_{ij}^{k}f_{mn}^{k}}$$

(6) $$E(H_{k})={\bar{H}}_{k},\text{Cov}(h_{ij}^{k},h_{mn}^{k})=C_{h_{ij}^{k}h_{mn}^{k}}$$

Where $f_{ij}^{k}$ and $h_{ij}^{k}$ are the (*i*, *j*) *th* entries of matrices *F_k_* and *H_k_*, respectively.

Rewriting *F_k_*and *H_k_* as:

(7) $$F_{k}={\bar{F}}_{k}+{\tilde{F}}_{k}$$

(8) $$H_{k}={\bar{H}}_{k}+{\tilde{H}}_{k}$$

And substituting (7), (8) into (1), (2) converts the original system to:

(9) $$x_{k+1}={\bar{F}}_{k}x_{k}+{\tilde{v}}_{k}$$

(10) $$y_{k}={\bar{H}}_{k}x_{k}+{\tilde{\omega }}_{k}$$

where

(11) $$\begin{array}{l} {\tilde{v}}_{k}=v_{k}+{\tilde{F}}_{k}x_{k}\\ {\tilde{\omega }}_{k}=\omega_{k}+{\tilde{H}}_{k}x_{k} \end{array}$$

System (9), (10) has deterministic parameter matrices, but the process noise and observation noise are dependent on the state. Therefore, this would not satisfy the well-known assumptions of the standard Kalman filtering apparently.

In the appendix, we give a detailed proof that system (9) and (10) satisfy all conditions of the standard Kalman filtering and derive the recursive state estimate of the new system as follows:

Theorem 1. The Linear Minimum Variance recursive state estimation of system (9), (10) is given by:

$$\begin{array}{l} x_{k+1|k+1}=x_{k+1|k}+K_{k+1}\left(y_{k+1}-{\bar{H}}_{k+1}x_{k+1|k}\right)\\ x_{k+1|k}={\bar{F}}_{k}x_{k/k}\\ {\text{K}}_{\text{k}+1}=P_{k+1|k}{\bar{H}}_{k+1}^{T}{\left({\bar{H}}_{k+1}P_{k+1|k}{\bar{H}}_{k+1}^{T}+R_{{\tilde{\omega }}_{k}}\right)}^{+}\\ P_{k+1|k}={\bar{F}}_{k}P_{k/k}{\bar{F}}_{k}^{T}+R_{{\tilde{v}}_{k}}\\ P_{k+1|k+1}=\left(I-K_{k+1}{\bar{H}}_{k+1}\right)P_{k+1|k}\\ R_{{\tilde{v}}_{k}}=R_{v_{k}}+E\left({\tilde{F}}_{k}E\left(x_{k}x_{k}^{T}\right){\tilde{F}}_{k}^{T}\right)\\ R_{{\tilde{\omega }}_{k}}=R_{\omega_{k}}+E\left({\tilde{H}}_{k}E\left(x_{k}x_{k}^{T}\right){\tilde{H}}_{k}^{T}\right)\\ E\left(x_{k+1}x_{k+1}^{T}\right)={\bar{F}}_{k}E\left(x_{k}x_{k}^{T}\right){\bar{F}}_{k}+E\left({\tilde{F}}_{k}E\left(x_{k}x_{k}^{T}\right){\tilde{F}}_{k}^{T}\right)+R_{v_{k}}\\ x_{0|0}=Ex_{0},P_{0|0}=\text{Var}\left(x_{0}\right),E\left(x_{0}x_{0}^{T}\right)=Ex_{0}Ex_{0}^{T}+P_{0|0} \end{array}$$

where the superscript “+” denotes Moore-Penrose pseudo inverse, *x_k_*_+1|_*_k_* denotes the one-step prediction of *x_k_*_+1_, *P_k_*_+1|_*_k_* denotes the covariance of *x_k_*_+1|_*_k_*, *x_k_*_+1|_*_k_*_+1_ denotes the update of *x_k_*_+1_ and *P_k_*_+1|_*_k_*_+1_ denotes the covariance of *x_k_*_+1|_*_k_*_+1_.

Compared with the standard Kalman filtering and noting the notations in (5), (6), the random parameter matrices Kalman filtering has one more recursion of $E(x_{k+1}x_{k+1}^{T})$ as follows:

$$E\left(x_{k+1}x_{k+1}^{T}\right)={\bar{F}}_{k}E\left(x_{k}x_{k}^{T}\right){\bar{F}}_{k}+E\left({\tilde{F}}_{k}E\left(x_{k}x_{k}^{T}\right){\tilde{F}}_{k}^{T}\right)+R_{v_{k}}$$

where

$$\begin{array}{c} E{\left({\tilde{F}}_{k}E\left(x_{k}x_{k}^{T}\right){\tilde{F}}_{k}^{T}\right)}_{\left(m,n\right)}=\overset{r}{\underset{i=1}{\text{∑}}}C_{f_{n1}^{k}f_{mi}^{k}}X_{i1}^{k}+\ldots +\overset{r}{\underset{i=1}{\text{∑}}}C_{f_{nr}^{k}f_{mi}^{k}}X_{ir}^{k}\\ E{\left({\tilde{H}}_{k}E\left(x_{k}x_{k}^{T}\right){\tilde{H}}_{k}^{T}\right)}_{\left(m,n\right)}=\overset{r}{\underset{i=1}{\text{∑}}}C_{h_{n1}^{k}h_{mi}^{k}}X_{i1}^{k}+\ldots +\overset{r}{\underset{i=1}{\text{∑}}}C_{h_{nr}^{k}h_{mi}^{k}}X_{ir}^{k} \end{array}$$

and where $X_{ij}^{k}$ is the (*i*, *j*) *th* entries of $X^{k}=E\left(x_{k}x_{k}^{T}\right)$.

## 3. Random Parameter Matrices Kalman Filtering with Multisensor Fusion

In this section, a new distributed Kalman filtering fusion with random parameter matrices is proposed. The framework of the distributed tracking system is the same as those considered in [12-15]. The advantages of transmitting sensor estimates other than sensor measurements can be seen in [12-15]. We will show that under a mild condition the fused state estimate is equivalent to the centralized Kalman filtering using all sensor measurements. Therefore, it achieves the best performance.

The *l*-sensor dynamic system is given by:

(12) $$\begin{array}{ll} x_{k+1}=F_{k}x_{k}+v_{k}, & k=0,1,\ldots \\ y_{k}^{i}=H_{k}^{i}x_{k}+\omega_{k}^{i} & i=1,\ldots ,l \end{array}$$

where *x_k_* ∈ *R^r^* is the state, $y_{k}^{i}\in R^{N^{i}}$ is the measurement matrix in *i*-th sensor, *v_k_* ∈ *R^r^* is the process noise, and $\omega_{k}^{i}\in R^{N^{i}}$is the measurement noise. Parameter matrices *F_k_* and $H_{k}^{i}$ are random. We assume that $\left\{F_{k},H_{k}^{i},v_{k},\omega_{k}^{i},k=0,1,2\ldots \right\}$, *i*, *j* =, …*l*, is a sequence of independent variables. Every single sensor satisfies the assumption in the last section.

Convert system (12) to the following one with deterministic parameter matrices:

(13) $$\begin{array}{ll} x_{k+1}={\bar{F}}_{k}x_{k}+{\tilde{v}}_{k}, & k=0,1,\ldots \\ y_{k}^{i}={\bar{H}}_{k}^{i}x_{k}+{\tilde{\omega }}_{k}^{i} & i=1,\ldots ,l \end{array}$$

where

$$\begin{array}{l} {\tilde{v}}_{k}=v_{k}+{\tilde{F}}_{k}x_{k}\\ {\tilde{\omega }}_{k}^{i}=\omega_{k}+{\tilde{H}}_{k}^{i}x_{k} \end{array}$$

The stacked measurement equation is written as:

$$y_{k}={\bar{H}}_{k}x_{k}+{\tilde{\omega }}_{k}$$

where

$$y_{k}={\left(y_{k}^{1^{T}},\ldots ,y_{k}^{l^{T}}\right)}^{T},{\bar{H}}_{k}={\left({\bar{H}}_{k}^{1^{T}},\ldots ,{\bar{H}}_{k}^{l^{T}}\right)}^{T},{\tilde{\omega }}_{k}={\left({\tilde{\omega }}_{k}^{1^{T}},\ldots ,{\tilde{\omega }}_{k}^{l^{T}}\right)}^{T}$$

and the covariance of the noise *ω̃_k_* is given by:

$$\text{Var}\left({\tilde{\omega }}_{k}\right)={\tilde{R}}_{k}$$

Consider the covariance of the measurement noise of single sensor in new system. By the assumption above, we have:

$$\begin{array}{l} E\left({\tilde{\omega }}_{k}^{i}{\tilde{\omega }}_{k}^{jT}\right)=E\left(\omega_{k}^{i}+{\tilde{H}}_{k}^{i}x_{k}\right){\left(\omega_{k}^{j}+{\tilde{H}}_{k}^{j}x_{k}\right)}^{T}\\ =E\left({\tilde{\omega }}_{k}^{i}{\tilde{\omega }}_{k}^{iT}+\omega_{k}^{i}x_{k}^{T}{\tilde{H}}_{k}^{jT}+{\tilde{H}}_{k}^{i}x_{k}{\tilde{\omega }}_{k}^{jT}+{\tilde{H}}_{k}^{i}x_{k}x_{k}^{T}{\tilde{H}}_{k}^{jT}\right)\\ =E\left({\tilde{\omega }}_{k}^{i}{\tilde{\omega }}_{k}^{jT}\right)+E\left[{\tilde{H}}_{k}^{i}E\left(x_{k}x_{k}^{T}\right){\tilde{H}}_{k}^{jT}\right] \end{array}$$

As shown in the last part of Section 2, every entry of the last matrix term of the above equation is a linear combination of $E\left(h_{ij}^{k}h_{ls}^{k}\right)$. Hence, when ${\tilde{H}}_{k}^{i}$ and ${\tilde{H}}_{k}^{j}$ are correlated, in general, $E\left[{\tilde{H}}_{k}E\left(x_{k}x_{k}^{T}\right){\tilde{H}}_{k}^{jT}\right]\neq 0$. Therefore, even if $\left(\omega_{k}^{i}\omega_{k}^{iT}\right)=0$, i.e., the original sensor noises are mutually independent, the sensor noises of the converted system are still cross-correlated, i.e., R̃_k_ is non-diagonal block matrix.

Luckily, when sensor noises are cross-correlated, in [7], it was proven that under a mild condition the fuse state estimate is equivalent to the centralized Kalman filtering using all sensor measurments.

According to Theorem 1 and the Kalman filtering formulae given in [8-10], the local Kalman filtering at the *i*-th sensor is:

(14) $$\begin{array}{l} x_{k|k}^{i}=x_{k|k-1}^{i}+K_{k}^{i}\left(y_{k}^{i}-{\bar{H}}_{k}x_{k|k-1}^{i}\right)=\left(I-K_{k}^{i}{\bar{H}}_{k}^{i}\right)x_{k|k-1}^{i}+K_{k}^{i}y_{k}^{i}\\ K_{k}^{i}=P_{k|k}^{i}{\bar{H}}_{k}^{iT}{\tilde{R}}_{k}^{i-1} \end{array}$$

where

$${\tilde{R}}_{k}^{i}=\text{Var}\left({\tilde{\omega }}_{k}^{i}\right)$$

with covariance of filtering error given by:

$$P_{k|k}^{i}=\left(I-K_{k}^{i}{\bar{H}}_{k}^{i}\right)P_{k|k-1}^{i}$$

or

(15) $$P_{k|k}^{i^{-1}}=P_{k|k}^{i^{-1}}+{\bar{H}}_{k}^{i^{T}}{\tilde{R}}_{k}^{i^{-1}}{\bar{H}}_{k}^{i}$$

where

$$\begin{array}{l} x_{k|k-1}^{i}={\bar{F}}_{k}x_{k-1|k-1}^{i}\\ p_{k|k}^{i}=E\left(x_{k|k}^{i}-x_{k}\right){\left(x_{k|k}^{i}-x_{k}\right)}^{T}\\ p_{k|k-1}^{i}=E\left(x_{k|k-1}^{i}-x_{k}\right){\left(x_{k|k}^{i}-x_{k}\right)}^{T} \end{array}$$

We assume that the system has the following properties: the row dimensions of all sensor measurement matrices ${\bar{H}}_{k}^{i}$ to be less than or equal to the dimension of the state, and all ${\bar{H}}_{k}^{i}$ to be of full row rank. In many practical applications, this assumption is fulfilled very often. Thus, we know ${\bar{H}}_{k}^{i}{\left({\bar{H}}_{k}^{i}\right)}^{+}=I$.

According to [7] and Theorem 1, the centralized Kalman filtering with all sensor data is given by:

(16) $$\begin{array}{l} x_{k|k}=x_{k|k-1}+K_{k}\left(y_{k}-{\bar{H}}_{k}x_{k|k-1}\right)\\ =\left(I-K_{k}{\bar{H}}_{k}\right)x_{k|k-1}+K_{k}y_{k}\\ =\left(I-K_{k}{\bar{H}}_{k}\right)x_{k|k-1}+P_{k|k}{\bar{H}}_{k}^{T}\sum_{i=1}^{l}{\tilde{R}}_{k}^{-1}(*i)y_{k}^{i} \end{array}$$

(17) $$K_{k}=P_{k|k}{\bar{H}}_{k}^{T}{\tilde{R}}_{k}^{-1}$$

where, ${\tilde{R}}_{k}^{-1}\left(*i\right)$ is the *i*-th column block of ${\tilde{R}}_{k}^{-1}$. The covariance of filtering error given by:

(18) $$\begin{array}{l} P_{k|k}^{-1}=P_{k|k-1}^{-1}+{\bar{H}}_{k}^{T}{\tilde{R}}_{k}^{-1}{\bar{H}}_{k}\\ =P_{k|k}^{-1}+{\bar{H}}_{k}^{T}\overset{l}{\underset{i=1}{\text{∑}}}{\tilde{R}}_{k}^{-1}\left(*i\right){\tilde{R}}_{k}^{i}{\left({\bar{H}}_{k}^{i^{T}}\right)}^{+}{\bar{H}}_{k}^{i^{T}}{\tilde{R}}_{k}^{i^{-1}}{\bar{H}}_{k}^{i} \end{array}$$

or

(19) $$P_{k|k}=\left(I-K_{k}{\bar{H}}_{k}\right)P_{k|k-1}$$

Where

$$\begin{array}{l} x_{k|k-1}={\bar{F}}_{k}x_{k-1|k-1}\\ P_{k|k}=E\left(x_{k|k}-x_{k}\right){\left(x_{k|k}-x_{k}\right)}^{T}\\ P_{k|k-1}=E\left(x_{k|k-1}-x_{k}\right){\left(x_{k|k-1}-x_{k}\right)}^{T} \end{array}$$

Using (15) and (18), the estimation error covariance of the centralized Kalman filtering is given by using the estimation error covariance of all local filters:

(20) $$P_{k|k}^{-1}=P_{k|k-1}^{-1}+{\bar{H}}_{k}^{T}\overset{l}{\underset{i=1}{\text{∑}}}{\tilde{R}}_{k}^{-1}(*i){\tilde{R}}_{k}^{i}{\left({\tilde{H}}_{k}^{i^{T}}\right)}^{+}\left(P_{k|k}^{i^{-1}}-P_{k|k-1}^{i^{-1}}\right)$$

Using (17):

(21) $$\begin{array}{l} K_{k}y_{k}=P_{k|k}{\bar{H}}_{k}^{T}\overset{l}{\underset{i=1}{\text{∑}}}{\tilde{R}}_{k}^{-1}(*i)y_{k}^{i}\\ =P_{k|k}{\bar{H}}_{k}^{T}\overset{l}{\underset{i=1}{\text{∑}}}{\tilde{R}}_{k}^{-1}(*i){\tilde{R}}_{k}^{i}{\left({\bar{H}}_{k}^{i^{T}}\right)}^{+}{\bar{H}}_{k}^{i^{T}}{\tilde{R}}_{k}^{i^{-1}}y_{k}^{i} \end{array}$$

To express the centralized filtering *x_k_*_|_*_k_* in terms of the local filtering, by (14) and (15), we have

(22) $$\begin{array}{l} {\bar{H}}_{k}^{i^{T}}{\tilde{R}}_{k}^{i^{-1}}y_{k}^{i}=P_{k|k}^{i^{-1}}K_{k}^{i}y_{k}^{i}\\ =P_{k|k}^{i^{-1}}\left[x_{k|k}^{i}-\left(I-K_{k}^{i}{\bar{H}}_{k}^{i}\right)x_{k|k-1}^{i}\right]\\ =P_{k|k}^{i^{-1}}x_{k|k}^{i}-P_{k|k-1}^{i^{-1}}x_{k|k-1}^{i} \end{array}$$

Thus, substituting (19), (21) and (22) into (16) yields:

(23) $$P_{k|k}^{-1}x_{k|k}=P_{k|k-1}^{-1}x_{k|k-1}+{\bar{H}}_{k}^{T}\overset{l}{\underset{i=1}{\text{∑}}}{\tilde{R}}_{k}^{-1}(*i){\tilde{R}}_{k}^{i}{\left({\bar{H}}_{k}^{i^{T}}\right)}^{+}\left(P_{k|k}^{i^{-1}}x_{k|k}^{i}-P_{k|k-1}^{i^{-1}}x_{k|k-1}^{i}\right)$$

That is to say that the centralized filtering (23) and error matrix (20) are explicitly expressed in terms of the local filtering. Hence, the performance of the distributed random parameter matrices Kalman filtering fusion is the same as that of the centralized random parameter matrices fusion.

### Remark 1

In this paper it is assumed that all sensor observations are synchronous. In practice, this may be very rarely true. However, in the past 20 years, such assumption was used very often in the track fusion community (for example, see [7, 11-13] among others). One of reasons for this is that it is easy to extend the results for synchronous distributed Kalman filtering fusion to the corresponding asynchronous case by letting $x_{k|k}^{i}=x_{k|k-1}^{i}$ and $P_{k|k}^{i}=P_{k|k-1}^{i}$ when the *i* th sensor does not receive its observation $y_{k}^{i}$ at time *k* to reduce the asynchronous distributed tracking system to be synchronous.

### Remark 2

The distributed systems here and in [7, 11-15] have a fusion center and the local sensor should transmit $x_{k|k}^{i},x_{k|k-1}^{i}P_{k|k}^{i},P_{k|k-1}^{i}$ and $P_{k|k-1}^{i}$to the fusion center. Our purpose in this paper is to derive a globally optimal distributed fusion algorithm equivalent to the centralized Kalman filtering as the fusion center can receive all sensor observations. In this framework, the system has not only the global optimality, but also the good survivability in a poor situation. Clearly, such systems are different from the system considered [16]. The system in [16] does not require any form of central processing facility, and the algorithm there is highly resilient to loss of one or more sensing nodes, but costs more communication and has no real-time global optimality. Thus, both of them have their own advantages and disadvantages. The Random Kalman filtering in the framework of [16] may be another future research direction.

## 4. Applications of Random Parameter Matrices Kalman Filtering

In this section, we will see that the results in the last two sections can be applied to the Kalman filtering with uncertain observations as well as randomly variant dynamic systems with multiple models.

### 4.1. Application to a General Uncertain Observation

The Kalman filtering with uncertain observation attracted extensive attention [2-4]. There are two types of uncertain observations in practice. The first one is that the estimator can exactly know whether the observation fully or partially contains the signal to be estimated, or just contains noise alone (for example, see [2]). By directly using the optimal estimation theory, the Kalman filter for the first type of uncertain observations can be derived easily. The other uncertain observations belong to the second type, i.e., the estimator cannot know whether the observation fully or partially contains the signal to be estimated or just contains noise alone, but the occurrence probabilities of each case are known. Clearly, the latter is more practical. By applying the random measurement matrix Kalman filtering, we can derive the Kalman filter with the second type of uncertain observations, which is much more general than that in [2-4].

Consider a system:

(24) $$x_{k+1}=F_{k}x_{k}+v_{k}$$

(25) $$y_{k}=\overset{l}{\underset{i=`}{\text{∑}}}I_{\left\{y_{k}=i\right\}}H_{k}^{i}x_{k}+\overset{l}{\underset{i=1}{\text{∑}}}I_{\left\{y_{k}=i\right\}}\omega_{k}^{i}$$

where all the parameter matrices are non-random and a set of multiple observation equations is selected to represent the possible observation case at each time. The random variable *γ_k_* is either observable or unobservable. If *γ_k_* = *i*, the measure matrix is $H_{k}^{i}$ and the observation noise corresponds to $\omega_{k}^{i}$. When the value of *γ_k_* is observable at each time *k*, this is an uncertain observation of the first type and the state estimation with measurement Equation (25) is converted to:

(26) $$y_{k}=H_{k}^{i}x_{k}+\omega_{k}^{i}$$

which is obviously the classical Kalman filtering, i.e., the least mean square estimate using the various available observation of *y_k_*. To show the applications of the random measurement matrix Kalman filtering, we focus on the second type of uncertain observation, i.e., in (25), *γ_k_* is unobservable at each time *k*, but the probability of occurrence of every available measurement matrix is known.

Consider that in (25), *γ_k_* is unobservable at each time *k*, but the probability of the occurrence of each measurement is known. Obviously, (2) is a more general form of (25) because only expectation and covariance of *H_k_* in (2) are known other than its distribution. The expectation of *H_k_* can be expressed as:

(27) $${\bar{H}}_{k}=\overset{l}{\underset{j=1}{\text{∑}}}p_{k}^{j}H_{k}^{j}$$

(28) $${\tilde{H}}_{k}=H_{k}^{i}-{\bar{H}}_{k},\text{with probability}\;p_{k}^{i}$$

All that remains in order to apply the random measurement matrix Kalman filtering is just to calculate:

(29) $$R_{{\tilde{\omega }}_{k}}=R_{\omega_{k}}+E({\tilde{H}}_{k}E(x_{k}x_{k}^{T}){\tilde{H}}_{k}^{T})=R_{\omega_{k}}+\overset{l}{\underset{i=1}{\text{∑}}}p_{k}^{i}(H_{k}^{i}-{\bar{H}}_{k})E(x_{k}x_{k}^{T}){\left(H_{k}^{i}-{\bar{H}}_{k}\right)}^{T}$$

Substituting (27}) and (29) into Theorem 1 can immediately obtain the random measurement matrix Kalman filtering of model (1) and (25).

In the classical Kalman filtering problem, the observation is always assumed to contain the signal to be estimated. However, in practice, when the exterior interference is strong, i.e., total covariance of the measurement noise is large; the estimator will mistake the noise as the observation sometimes. In radar terminology, this is called a false alarm. Usually, the estimator cannot know whether this happens or not, only the probability of a false alarm is known. In the following, we will show that the Kalman Filtering with a false alarm probability is a special case of the uncertain observations of the above model (1), (25) are given.

Consider a discrete dynamic process:

(30) $$x_{k+1}=F_{k}x_{k}+v_{k}$$

(31) $$y_{k}=h_{k}x_{k}+\omega_{k},k=0,1,2\ldots$$

where {*F_k_*, *h_k_*, *v_k_*, *ω_k_*, *k* = 0, 1, 2, …} satisfy the assumptions of standard Kalman filtering. *F_k_* and *h_k_* are deterministic matrices. The false alarm probability of the observation is 1 – *p_k_*.

Then,we can rewrite the measurement equations as follows:

(32) $$y_{k}=H_{k}x_{k}+\omega_{k},k=0,1,2,\ldots$$

where the observation matrix *H_k_* is a binary-valued random with:

(33) $$\mathrm{Pr}\{H_{k}=h_{k}\}=p_{k}$$

(34) $$\mathrm{Pr}\{H_{k}=0\}=1-p_{k}$$

Due to (8):

(35) $${\bar{H}}_{k}=p_{k}h_{k}$$

(36) $$\mathrm{Pr}\{\tilde{H}=(1-p_{k})h_{k}\}=p_{k}$$

(37) $$\mathrm{Pr}\{\tilde{H}=-p_{k}h_{k}\}=1-p_{k}$$

In the false alarm case, the state transition matrix is still deterministic, but the measurement matrix is random, by (35), (36) and (37), the covariance of the process and observation noises can be written as follows:

(38) $$R_{{\tilde{v}}_{k}}=R_{v_{k}}$$

(39) $$R_{{\tilde{\omega }}_{k}}=R_{\omega_{k}}+E\left({\tilde{H}}_{k}E\left(x_{k}x_{k}^{T}\right){\tilde{H}}_{k}^{T}\right)=R_{\omega_{k}}+(1-p_{k})p_{k}h_{k}E(x_{k}x_{k}^{T})h_{k}^{T}$$

Thus, the Kalman filtering with false alarm probability in this case is given by:

$$\begin{array}{l} x_{k+1|k+1}=x_{k+1|k}+K_{k+1}(y_{k+1}-p_{k+1}h_{k+1}x_{k+1|k})\\ x_{k+1|k}=F_{k}x_{k|k}\\ K_{k+1}=p_{k+1}P_{k+1|k}h_{k+1}^{T}{\left(p_{k+1}^{2}h_{k+1}P_{k+1|k}h_{k+1}^{T}+R_{{\tilde{\omega }}_{k}}\right)}^{+}\\ P_{k+1|k}=F_{k}P_{k|k}F_{k}^{T}+R_{{\tilde{v}}_{k}}\\ P_{k+1|k+1}=(I-p_{k+1}K_{k+1}h_{k+1})P_{k+1|k}\\ R_{{\tilde{\omega }}_{k}}=R_{\omega_{k}}+(1-p_{k})p_{k}h_{k}E(x_{k}x_{k}^{T})h_{k}^{T}\\ E(x_{k+1}x_{k+1}^{T})=F_{k}E(x_{k}x_{k}^{T})F_{k}^{T}+R_{v_{k}}\\ x_{0|0}=Ex_{0},P_{0|0}=\text{Var}(x_{0}),E(x_{0}x_{0}^{T})=Ex_{0}Ex_{0}^{T}+P_{0|0} \end{array}$$

In this section, we consider the application to a general uncertain observation for one sensor case. In a manner analogous to the derivation of Section 4.1, we can also give an application to a general uncertain observation for multisensor case using Section 3. The procedure is omitted here.

### 4.2. Application to a Multi-Model Dynamic Process

The multiple-model (MM) dynamic process has been considered by many researchers. Although the possible models considered in those papers are quite general and can depend on the state, but no optimal algorithm in the mean square error (MSE) sense was proposed in the past a few decades. On the other hand, when some of the MM systems satisfy the assumptions in this paper, they can be reduced to dynamic models with random transition matrix and thus the optimal real-time filter can be given directly according to the random transition matrix Kalman filtering proposed in Theorem 1.

Consider a system:

(40) $$x_{k+1}=F_{k}^{i}x_{k}+v_{k}\;\text{with probability}\;p_{k}^{i},i=1,\ldots ,l$$

(41) $$y_{k}=H_{k}x_{k}+\omega_{k}$$

where *F_k_* and *v_k_* are independent sequence, and *H_k_* is non-random. We use random matrix *F_k_* to stand for the state transition matrix. The expectation of *F_k_* can be expressed as:

(42) $${\bar{F}}_{k}=\overset{l}{\underset{j=1}{\text{∑}}}p_{k}^{j}F_{k}^{j}$$

(43) $${\tilde{F}}_{k}=F_{k}^{i}-{\bar{F}}_{k},\text{with probability}\;p_{k}^{i}$$

A necessary step for implementing the random Kalman filtering is to calculate:

(44) $$R_{{\tilde{v}}_{k}}=R_{v_{k}}+E\left({\tilde{F}}_{k}E\left(x_{k}x_{k}^{T}\right){\tilde{F}}_{k}^{T}\right)=R_{v_{k}}+\overset{l}{\underset{i=1}{\text{∑}}}p_{k}^{i}\left(F_{k}^{i}-{\bar{F}}_{k}\right)E\left(x_{k}x_{k}^{T}\right){\left(F_{k}^{i}-{\bar{F}}_{k}\right)}^{T}$$

Thus, all the recursive formulas of random Kalman filtering can be given by:

$$\begin{array}{l} x_{k+1|k+1}=x_{k+1|k}+K_{k+1}(y_{k+1}-H_{k+1}x_{k+1|k})\\ x_{k+1|k}={\bar{F}}_{k}x_{k|k}\\ K_{k+1}=p_{k+1}P_{k+1|k}H_{k+1}^{T}{\left(H_{k+1}P_{k+1|k}H_{k+1}^{T}+R_{\omega_{k}}\right)}^{+}\\ P_{k+1|k}={\bar{F}}_{k}P_{k|k}{\bar{F}}_{k}^{T}+R_{{\tilde{v}}_{k}}\\ P_{k+1|k+1}=(I-K_{k+1}H_{k+1})P_{k+1|k}\\ R_{{\tilde{v}}_{k}}=R_{v_{k}}+\overset{l}{\underset{i=1}{\text{∑}}}p_{k}^{i}(F_{k}^{i}-{\bar{F}}_{k})E(x_{k}x_{k}^{T}){\left(F_{k}^{i}-{\bar{F}}_{k}\right)}^{T}\\ E(x_{k+1}x_{k+1}^{T})={\bar{F}}_{k}E(x_{k}x_{k}^{T}){\bar{F}}_{k}^{T}+R_{v_{k}}+\overset{l}{\underset{i=1}{\text{∑}}}p_{k}^{i}(F_{k}^{i}-{\bar{F}}_{k})E(x_{k}x_{k}^{T}){\left(F_{k}^{i}-\bar{F}\right)}^{T}\\ x_{0|0}=Ex_{0},P_{0|0}=\text{Var}(x_{0}),E(x_{0}x_{0}^{T})=Ex_{0}Ex_{0}^{T}+P_{0|0} \end{array}$$

## 5. Numerical Example

In this section, three simulations will be done for a dynamic system with random parameter matrices modeled as an object movement with process noise and measurement noise on the plane. The simulations give the special applications of results in the last section and show that fused random parameter matrices Kalman filtering algorithms can track the object satisfactorily.

Remember that we have rigorously proved in Section 3 that the centralized algorithm using all sensor observations $y_{k}^{i}$ at the fusion center can be equivalently converted to be distributed algorithm using all sensor estimates $x_{k|k}^{i}$. In addition, the computer simulations we have done show that the simulation results of two algorithms are exactly the same. It turns out that in the following numerical examples, we only compare the distributed random Kalman filtering and the corresponding standard Kalman filtering that ignores the randomness of the parameter matrices. Without loss of generality our examples assume the local sensors send updates each time when they receive a measurement.

Firstly, we consider a three-sensor distributed Kalman filtering fusion problem with false alarm probabilities.

### Example 1

The object dynamics and measurement equations are modeled as follows:

$$\begin{array}{l} x_{k+1}=F_{k}x_{k}+v_{k}\\ y_{k}^{i}=H_{k}^{i}x_{k}+\omega_{k}^{i},i=1,2,3 \end{array}$$

where $\left\{F_{k},H_{k}^{i},v_{k}\omega_{k}^{i}k=0,1,2,\ldots \right\}$ satisfy the assumptions of standard Kalman filtering. The state transition matrix *F_k_*

$$F_{k}=\left(\begin{array}{ll} \cos(2\pi /300) & \sin(2\pi /300)\\ -\sin(2\pi /300) & \cos(2\pi /300) \end{array}\right)$$

is a constant. The measurement matrix is given by:

$$H_{k}^{i}=\left(\begin{array}{cc} 1 & i\\ 1 & -i \end{array}\right),i=1,2,H_{k}^{3}=\left(\begin{array}{cc} 1 & 3\\ -1 & 3 \end{array}\right)$$

The false alarm probability of the *l*-th sensor is given by:

$$1-p_{k}^{1}=0.01,1-p_{k}^{2}=0.02,1-p_{k}^{3}=0.03$$

The initial state *x*_0_ = (50, 0), $P_{0|0}^{i}=I$. The covariance of the noises are diagonal, given by *R_v_* =1, *R_ωi_* = 2 , *i* = 1,2,3. Using a Monte-Carlo method of 50 runs, we can evaluate tracking performance of an algorithm by estimating the second moment of the tracking error, given by:

$$E_{k}^{2}=\frac{1}{50}\overset{50}{\underset{i=1}{\text{∑}}}{\left\|x_{k|k}^{\left(i\right)}-x_{k}\right\|}^{2}$$

**Figure 1.**
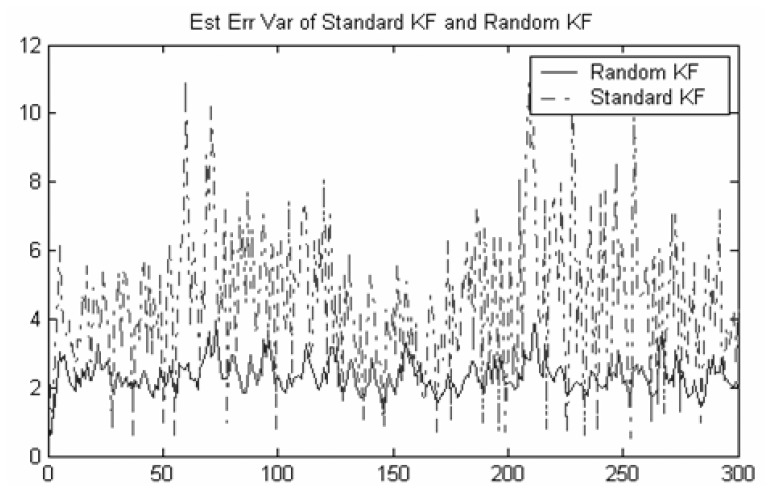
Comparison of standard Kalman filtering fusion and random Kalman filtering fusion.

Figure 1 shows that the second moments of tracking error for three sensors Kalman filtering fusion without considering the false alarm (i.e. standard Kalman filtering) and three sensors random Kalman filtering fusion considering the false alarm (i.e. random Kalman filtering), respectively. It can be shown that even if the false alarm probability is very small, the distributed Random Kalman filtering fusion performs much better than the standard Kalman filtering.

In Example 1, both the sensor noises and the random measure matrices of the original system are mutually independent, so the sensor noise of the converted system are mutually independent. Now, we consider another example that both the noises and the random measure matrices of the original system are cross-correlated.

### Example 2

The object dynamics and measurement equations are modeled as follows:

$$\begin{array}{c} x_{k+1}=F_{k}x_{k}+v_{k}\\ y_{k}^{i}=H_{k}^{i}x_{k}+\omega_{k}^{i},\text{with probability of}\;p_{k}\\ =\omega_{k}+\omega_{k}^{i},\text{with probability of}\;1-p_{k} \end{array}$$

where $i=1,2,3,\left\{F_{k},H_{k}^{i},v_{k},\omega_{k},\omega_{k}^{i}k=0,1,2,\ldots \right\}$ satisfy the assumptions of standard Kalman filtering. The state transition matrix *F_k_* and the measurement matrices $H_{k}^{i}$ are the same as Example 1 and *ω_k_* is a large transition noise. When it happens, the sensors will mistake the transition noise as the observation. The false alarm probability of the transition noise is given by1 − *p_k_* = 0.05. Though the sensor noises $\omega_{k}^{i},i=1,2,3$ are mutually independent and independent of *ω_k_*, but the total measurement noises ${\tilde{\omega }}_{k}^{i}$ are cross-correlated here. The covariance of the noises are diagonal, given by *R_v_* = 1, *R_ω_* = 2 *R_ωi_* = 0.5, *i* = 1,2,3. The initial state *x*_0_ = (50, 0), $P_{0|0}^{i}=I$.

**Figure 2.**
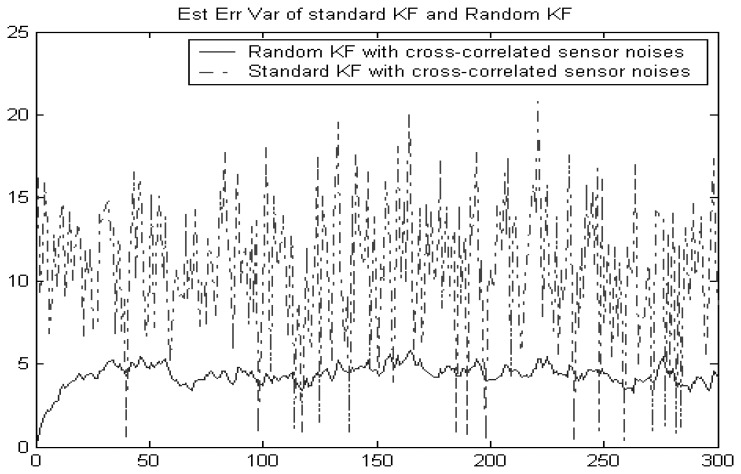
Comparison of standard Kalman filtering fusion and random Kalman filtering fusion.

In this example, both the measurement noises and the random measure matrices of the original system are cross-correlated. Hence, the sensor noises of the converted system are cross-correlated. Figure 2 shows that the random Kalman filtering fusion given in Section 3 still works better than the standard Kalman filtering without considering the false alarm. This implies that the standard Kalman filtering incorrectly assumes that sensor noises are independent.

### Example 3

In this simulation, there are three dynamic models with the corresponding probabilities of occurrence available. The object dynamics and measurement matrix in (40) are given by:

$$\begin{array}{l} F_{k}^{1}=\left(\begin{array}{ll} \cos(2\pi /300) & \sin(2\pi /300)\\ -\sin(2\pi /300) & \cos(2\pi /300) \end{array}\right)\text{with probability}\;0.1\\ F_{k}^{2}=\left(\begin{array}{ll} \cos(2\pi /250) & \sin(2\pi /250)\\ -\sin(2\pi /250) & \cos(2\pi /250) \end{array}\right)\text{with probability}\;0.2\\ F_{k}^{3}=\left(\begin{array}{ll} \cos(2\pi /100) & \sin(2\pi /50)\\ -\sin(2\pi /50) & \cos(2\pi /100) \end{array}\right)\text{with probability}\;0.1\\ H_{k}=\left(\begin{array}{cc} 1 & 1\\ 1 & -1 \end{array}\right) \end{array}$$

The covariance of the noises are diagonal, given by *R_v_* = 2, *R_ω_* = 1. In the following, we compare our numerical results with the IMM. Since in this example, the occurrence probability of each model at every time *k* is known and mutually independent, it is also the transition probability in the IMM. Therefore, the transition probability matrix ∏ at each time in the IMM is fixed and given by:

$$\prod =\left(\begin{array}{ccc} 0.1 & 0.2 & 0.7\\ 0.1 & 0.2 & 0.7\\ 0.1 & 0.2 & 0.7 \end{array}\right)$$

Π(*i*, *j*) here means the transition probability of model *i* to model *j* . This assumption also implies that the model probability in the IMM is fixed as follows:

$$\begin{array}{llll} \pi_{k}^{1}=0.1, & \pi_{k}^{2}=0.1, & \pi_{k}^{3}=0.7, & k=1,2\ldots \end{array}$$

**Figure 3.**
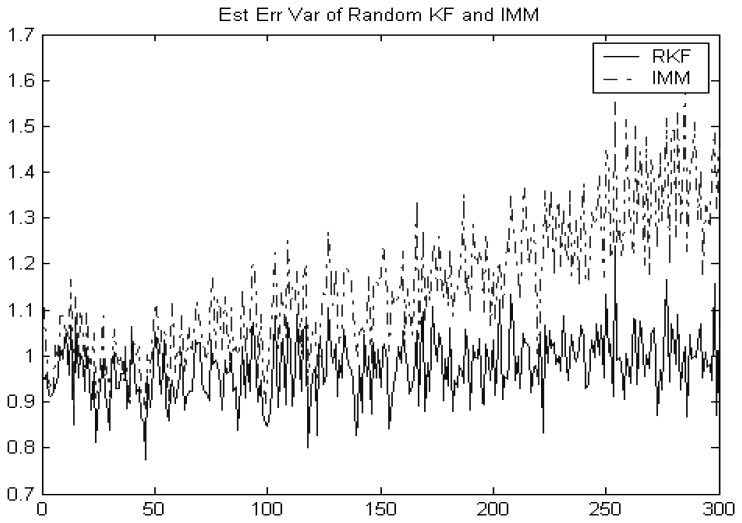
Comparison of IMM and random Kalman filtering.

Figure 3 shows that the random Kalman filtering given in section 4.2 still works better than the IMM with the fixed transition probability and model probability. This makes sense since the former is optimal in the MSE sense but the latter is not. However, in practice, the occurrence probability of each model is very often dependent on state or observation, and therefore not independent of each other in time. In this case, our new method offers no advantage.

### 6. Conclusions

In the multisensor random parameter matrices case, it was proven in this paper that when the sensor noises, or the measurement matrices of the original system are correlated across sensors, the sensor noises of the converted system are cross-correlated. Hence, such multisensor system seems not to satisfy the conditions for the standard distributed Kalman filtering fusion. This paper propose a new distributed Kalman filtering fusion with random parameter matrices Kalman filtering and proves that under a mild condition the fused state estimate is equivalent to the centralized Kalman filtering using all sensor measurements, therefore, it achieves the best performance. More importantly, this result can be applied to Kalman filtering with uncertain observations as well as randomly variant dynamic systems with multiple models. The Kalman filtering with false alarm is a special case of Kalman filtering with uncertain observations. Numerical examples are given which support our analysis and show significant performance loss of ignoring the randomness of the parameter matrices.

## Appendix

In the Appendix, we will give the proof of Theorem 1. Firstly, we give a detailed proof that system (9) and (10) satisfy all conditions of the standard Kalman filtering as follows:

### Lemma 1

Suppose random matrix *F* and random vector *x* are independent, then:

$$E\left(Fxx^{T}F^{T}\right)=E\left(FE\left(xx^{T}\right)F^{T}\right)$$

### Proof

By the properties of conditional expectation, we have that:

$$\begin{array}{l} E\left(Fxx^{T}F^{T}\right)=E\left(E\left(Fxx^{T}F^{T}|F\right)\right)\\ =E\left(FE\left(xx^{T}|F\right)F^{T}\right)\\ =E\left(FE\left(xx^{T}\right)F^{T}\right) \end{array}$$

**Q.E.D**.

### Lemma 2

$$\begin{array}{llll} (a)E({\tilde{v}}_{k})=0,E({\tilde{\omega }}_{k})=0; & & & \\ (b1)E(x_{0}{\tilde{v}}_{k}^{T})=0, & (b2)E(x_{0}{\tilde{\omega }}_{k}^{T})=0 & & \\ (c1)E({\tilde{v}}_{k}{\tilde{v}}_{l}^{T})=0, & (c2)E({\tilde{\omega }}_{k}{\tilde{\omega }}_{l}^{T})=0, & (c3)E({\tilde{v}}_{k}{\tilde{\omega }}_{l}^{T})=0, & \forall k\neq l\\ (d)E({\tilde{v}}_{k}{\tilde{v}}_{l}^{T})=R_{{\tilde{v}}_{k}},E({\tilde{\omega }}_{k}{\tilde{\omega }}_{l}^{T})=R_{\omega_{k}}, & & & \end{array}$$

where

$$\begin{array}{l} R_{{\tilde{v}}_{k}}=R_{v_{k}}+E\left({\tilde{F}}_{k}E\left(x_{k}x_{k}^{T}\right){\tilde{F}}_{k}^{T}\right),\\ R_{{\tilde{\omega }}_{k}}=R_{\omega_{k}}+E\left({\tilde{H}}_{k}E\left(x_{k}x_{k}^{T}\right){\tilde{H}}_{k}^{T}\right). \end{array}$$

### Proof

(a): By the assumptions on the model (1) and (2) , and notations in (11), it is obvious.
(b1): Since {*F_k_*, *v_k_*, *k* = 0,1,2,…} is independent of *x*_0_,
  $$\begin{array}{l} E(x_{0}{\tilde{v}}_{k}^{T})=E\left\{x_{0}{\left(v_{k}+{\tilde{F}}_{k}x_{k}\right)}^{T}\right\}\\ =E(x_{0}x_{k}^{T}{\tilde{F}}_{k}^{T})\\ =E\left\{x_{0}{\left(F_{k-1}x_{k-1}+v_{k-1}\right)}^{T}{\tilde{F}}_{k}^{T}\right\}\\ =E\left(x_{0}x_{k-1}^{T}F_{k-1}^{T}{\tilde{F}}_{k}^{T}\right)\\ =E\left\{x_{0}{\left(F_{k-2}x_{k-2}+v_{k-2}\right)}^{T}F_{k-1}^{T}{\tilde{F}}_{k}^{T}\right\}\\ =E\left(x_{0}x_{k-2}^{T}F_{k-2}^{T}F_{k-1}^{T}{\tilde{F}}_{k}^{T}\right)\\ \ldots \\ =E\left(x_{0}x_{0}^{T}F_{0}^{T}F_{1}^{T}\ldots F_{k-2}^{T}F_{k-1}^{T}{\tilde{F}}_{k}^{T}\right)\\ =E\left(x_{0}x_{0}^{T}\right)E\left(F_{0}^{T}\right)E\left(F_{1}^{T}\right)\ldots E\left(F_{k-2}^{T}\right)E\left(F_{k-1}^{T}\right)E\left({\tilde{F}}_{k}^{T}\right)\\ =0. \end{array}$$
(b2): Similarly, $E\left(x_{0}{\tilde{\omega }}_{k}^{T}\right)=0$.
(c1): Without loss of generality, we consider the case of *k* > *l* only.
  $$E\left({\tilde{v}}_{k}{\tilde{v}}_{l}^{T}\right)=E\left(v_{k}v_{l}^{T}+v_{k}x_{l}^{T}{\tilde{F}}_{l}^{T}+{\tilde{F}}_{k}x_{k}v_{l}^{T}+{\tilde{F}}_{k}x_{k}x_{l}^{T}{\tilde{F}}_{l}^{T}\right)$$
  For *x_l_* is linearly dependent on *F_l_*_−1_*F_l_*_−_*_2_*…*F*_1_*F*_0_*x*_0_ ,*v_l_*_−1_*,F_l_*_−1,_…*F_l_*_−_*_i_*_+1_*v_l_*_−_*_i,_i* = 2,3,…,*l*, and {*F_k,_v_k_*, *k* = 0,1,2,…}is independent of *x*_0_,
  $$\begin{array}{l} E(v_{k}x_{l}^{T}{\tilde{F}}_{l}^{T})=0\\ E\left({\tilde{F}}_{k}x_{k}v_{l}^{T}\right)=E\left\{{\tilde{F}}_{k}\left(F_{k-1}x_{k-1}+v_{k-1}\right)v_{l}^{T}\right\}\\ =E\left({\tilde{F}}_{k}F_{k-1}x_{k-1}v_{l}^{T}\right)\\ =E\left\{{\tilde{F}}_{k}F_{k-1}\left(F_{k-2}x_{k-2}+v_{k-2}\right)v_{l}^{T}\right\}\\ =E\left({\tilde{F}}_{k}F_{k-1}F_{k-2}x_{k-2}v_{l}^{T}\right)+E\left({\tilde{F}}_{k}F_{k-1}v_{k-2}v_{l}^{T}\right)\\ \ldots \\ =E\left({\tilde{F}}_{k}F_{k-1}\ldots F_{l}x_{l}V_{l}^{T}\right)+E\left({\tilde{F}}_{k}F_{k-1}\ldots F_{l+1}v_{l}v_{l}^{T}\right)\\ =E\left({\tilde{F}}_{k}\right)E\left(F_{k-1}\right)\ldots E\left(F_{l+1}\right)E\left(v_{l}v_{l}^{T}\right)\\ =0. \end{array}$$
  Noting that *xl* and {*F_k_* ,*k* = 0,1,2,…} are independent, by Lemma 1 we have:
  $$\begin{array}{l} E\left({\tilde{F}}_{k}x_{k}x_{l}^{T}{\tilde{F}}_{l}^{T}\right)=E\left\{{\tilde{F}}_{k}\left(F_{k-1}x_{k-1}+v_{k-1}\right)x_{l}^{T}{\tilde{F}}_{l}^{T}\right\}\\ =E\left({\tilde{F}}_{k}F_{k-1}x_{k-1}x_{l}^{T}{\tilde{F}}_{l}^{T}\right)\\ =E\left({\tilde{F}}_{k}F_{k-1}F_{k-2}x_{k-2}x_{l}^{T}{\tilde{F}}_{l}^{T}+{\tilde{F}}_{k}F_{k-1}v_{k-2}x_{l}^{T}{\tilde{F}}_{l}^{T}\right)\\ \ldots \\ =E\left({\tilde{F}}_{k}F_{k-1}F_{k-2}\ldots F_{l+1}F_{l}x_{l}x_{l}^{T}{\tilde{F}}_{l}^{T}\right)+E\left({\tilde{F}}_{k}F_{k-1}F_{k-2}\ldots F_{l+1}v_{l}x_{l}^{T}{\tilde{F}}_{l}^{T}\right)\\ =E\left({\tilde{F}}_{k}F_{k-1}F_{k-2}\ldots F_{l+1}{\tilde{F}}_{l}x_{l}x_{l}^{T}{\tilde{F}}_{l}^{T}\right)+E\left({\tilde{F}}_{k}F_{k-1}F_{k-2}\ldots F_{l+1}{\tilde{F}}_{l}x_{l}x_{l}^{T}{\tilde{F}}_{l}^{T}\right)\\ =E\left(E\left({\tilde{F}}_{k}F_{k-1}F_{k-2}\ldots F_{l+1}\right){\tilde{F}}_{l}E\left(x_{l}x_{l}^{T}\right){\tilde{F}}_{l}^{T}\right)\\ =0. \end{array}$$
  Hence, $E\left({\tilde{v}}_{k}{\tilde{v}}_{l}^{T}\right)=0$.
(c2): Also consider the case of *k*>*l* only. Since *x_l_* is linearly dependent on *F_l_*_−1_*F_l_*_−2_…*F*_1_*F*_0_*x*_0_,*v_l_*_−1_,*F_l_*_−1_*F_l_*_−2_…*F_l_*_−_*_i_*_+1_*v_l_*_−i_,*i*=2,3,…,*l*,{*F_k_, H_k_, v_k_*ω*_k_*=0,1,2,…} is independent of *x*_0_, and {*F_k_*, *H_k_*, *k* = 0,1,2,…} is independent of *x_l_* .Moreover:
  $$\begin{array}{l} E\left({\tilde{\omega }}_{k}{\tilde{\omega }}_{l}^{T}\right)=E\left(\omega_{k}\omega_{l}^{T}+\omega_{k}x_{l}^{T}{\tilde{H}}_{l}^{T}+{\tilde{H}}_{k}x_{k}\omega_{l}^{T}+{\tilde{H}}_{k}x_{k}x_{l}^{T}{\tilde{H}}_{l}^{T}\right)\\ =E\left({\tilde{H}}_{k}x_{k}x_{l}^{T}{\tilde{H}}_{l}^{T}\right)\\ =E\left\{{\tilde{H}}_{k}\left(F_{k-1}x_{k-1}+v_{k-1}\right)x_{l}^{T}{\tilde{H}}_{l}^{T}\right\}\\ =E\left({\tilde{H}}_{k}F_{k-1}x_{k-1}x_{l}^{T}{\tilde{H}}_{l}^{T}\right)\\ =E\left({\tilde{H}}_{k}F_{k-1}F_{k-2}x_{k-2}x_{l}^{T}{\tilde{H}}_{l}^{T}+{\tilde{H}}_{k}F_{k-1}v_{k-2}x_{l}^{T}{\tilde{H}}_{l}^{T}\right)\\ \ldots \\ =E\left({\tilde{H}}_{k}F_{k-1}F_{k-2}\ldots F_{l+1}F_{l}x_{l}x_{l}^{T}{\tilde{H}}_{l}^{T}\right)+E\left({\tilde{H}}_{k}F_{k-1}F_{k-2}\ldots F_{l+1}F_{l}v_{l}x_{l}^{T}{\tilde{H}}_{l}^{T}\right)\\ =0. \end{array}$$
(c3): $$E\left({\tilde{v}}_{k}{\tilde{\omega }}_{l}^{T}\right)=E\left(v_{k}\omega_{l}^{T}+v_{k}x_{l}^{T}{\tilde{H}}_{l}^{T}+{\tilde{F}}_{k}x_{k}\omega_{l}^{T}+{\tilde{F}}_{k}x_{k}x_{l}^{T}{\tilde{H}}_{l}^{T}\right).$$
  When *k*>*l*,
  $$\begin{array}{l} E\left({\tilde{v}}_{k}{\tilde{\omega }}_{l}^{T}\right)=E\left({\tilde{F}}_{k}x_{k}x_{l}^{T}{\tilde{H}}_{l}^{T}\right)\\ =E\left\{{\tilde{F}}_{k}\left(F_{k-1}x_{k-1}+v_{k-1}\right)x_{l}^{T}{\tilde{H}}_{l}^{T}\right\}\\ =E\left({\tilde{F}}_{k}F_{k-1}x_{k-1}x_{l}^{T}{\tilde{H}}_{l}^{T}\right)\\ =E\left({\tilde{F}}_{k}F_{k-1}F_{k-2}x_{k-1}x_{l}^{T}{\tilde{H}}_{l}^{T}\right)+E\left({\tilde{F}}_{k}F_{k-1}v_{k-2}x_{l}^{T}{\tilde{H}}_{l}^{T}\right)\\ \ldots \\ =E\left({\tilde{F}}_{k}F_{k-1}F_{k-2}\ldots F_{l+1}F_{l}x_{l}x_{l}^{T}{\tilde{H}}_{l}^{T}+{\tilde{F}}_{k}F_{k-1}F_{k-2}\ldots F_{l+1}v_{l}x_{l}^{T}{\tilde{H}}_{l}^{T}\right)\\ =0. \end{array}$$
  when *k*<*l*,
  $$\begin{array}{l} E\left(v_{k}x_{l}^{T}{\tilde{H}}_{l}^{T}\right)=E\left\{v_{k}{\left(F_{l-1}x_{l-1}+v_{l-1}\right)}^{T}{\tilde{H}}_{l}^{T}\right\}\\ =E\left(v_{k}x_{l-1}^{T}F_{l-1}^{T}{\tilde{H}}_{l}^{T}\right)\\ =E\left(v_{k}x_{l-2}^{T}F_{l-2}^{T}{\tilde{H}}_{l}^{T}\right)+E\left(v_{k}v_{l-2}^{T}F_{l-1}^{T}{\tilde{H}}_{l}^{T}\right)\\ \ldots \\ =E\left(v_{k}x_{k}^{T}F_{k}^{T}F_{k+2}^{T}\ldots F_{l-1}^{T}{\tilde{H}}_{l}^{T}\right)+E\left(v_{k}v_{k}^{T}F_{k+2}^{T}\ldots F_{l-1}^{T}{\tilde{H}}_{l}^{T}\right)\\ =0, \end{array}$$
  $$\begin{array}{l} E\left({\tilde{F}}_{k}x_{k}x_{l}^{T}{\tilde{H}}_{l}^{T}\right)=E\left\{{\tilde{F}}_{k}x_{k}{\left(F_{l-1}x_{l-1}+v_{l-1}\right)}^{T}{\tilde{H}}_{l}^{T}\right\}\\ =E\left({\tilde{F}}_{k}x_{k}x_{l-1}^{T}F_{l-1}^{T}{\tilde{H}}_{l}^{T}\right)\\ =E\left\{{\tilde{F}}_{k}x_{k}{\left(F_{l-2}x_{l-2}+v_{l-2}\right)}^{T}F_{l-1}^{T}{\tilde{H}}_{l}^{T}\right\}\\ \ldots \\ E\left\{{\tilde{F}}_{k}x_{k}{\left(F_{k}x_{k}+v_{k}\right)}^{T}F_{k+1}^{T}\ldots F_{l-1}^{T}{\tilde{H}}_{l}^{T}\right\}\\ =E\left({\tilde{F}}_{k}x_{k}x_{k}^{T}F_{k}^{T}F_{k+1}^{T}\ldots F_{l-1}^{T}{\tilde{H}}_{l}^{T}\right)\\ =E\left({\tilde{F}}_{k}E\left(x_{k}x_{k}^{T}\right)F_{k}^{T}E\left(F_{k+1}^{T}\ldots F_{l-1}^{T}{\tilde{H}}_{l}^{T}\right)\right)\\ =0 \end{array}$$
  Thus, $E\left({\tilde{v}}_{k}{\tilde{\omega }}_{l}^{T}\right)=0$.
(d): $$\begin{array}{l} E\left({\tilde{v}}_{k}{\tilde{v}}_{k}^{T}\right)=E\left(v_{k}v_{k}^{T}+v_{k}x_{k}^{T}{\tilde{F}}_{k}^{T}+{\tilde{F}}_{k}x_{k}v_{k}^{T}+{\tilde{F}}_{k}x_{k}x_{k}^{T}{\tilde{F}}_{k}^{T}\right)\\ =R_{v_{k}}+E\left({\tilde{F}}_{k}x_{k}x_{k}^{T}{\tilde{F}}_{k}^{T}\right)\\ =R_{v_{k}}+E\left({\tilde{F}}_{k}E\left(x_{k}x_{k}^{T}\right){\tilde{F}}_{k}^{T}\right) \end{array}$$
  Similarly, $E\left({\tilde{\omega }}_{k}{\tilde{\omega }}_{l}^{T}\right)=R_{\omega_{k}}+E\left({\tilde{H}}_{k}E\left(x_{k}x_{k}^{T}\right){\tilde{H}}_{k}^{T}\right)$.
  **Q.E.D.**
  By Lemma 2, system (9), (10) satisfies all conditions of the standard Kalman Filtering. Hence, we derive the recursive state estimate of the new system, i.e., Theorem 1 immediately.
